# Sonographic assessment of Achilles tendon in patients with cutaneous psoriasis 

**DOI:** 10.22088/cjim.15.2.313

**Published:** 2024

**Authors:** Anwar Sharifaskari, Azadeh Goodarzi, Navid Davoody, Nahid Kianmehr, Ali Sepyani, Anousheh Haghighi

**Affiliations:** 1 Department of Internal Medicine, School of Medicine, Iran University of Medical Sciences, Tehran, Iran; 2 Department of Dermatology, Rasoul Akram Medical Complex Clinical Research Development Center (RCRDC), School of Medicine, Iran University of Medical Sciences, Tehran, Iran; 3 Azad University of Medical Sciences

**Keywords:** Cutaneous psoriasis, Achilles tendon, Ultrasound, Enthesis, Psoriatic arthritis

## Abstract

**Background:**

Psoriasis is a common, chronic, immune-‌‌‌‌‌‌mediated inflammatory disease with a variety of skin manifestations. The aim of this study was to determine the prevalence of subclinical Achilles tendon disorder in cutaneous psoriasis patients and compare it with healthy controls.

**Methods:**

This was a cross-sectional case-control study conducted on psoriasis patients that were referred to dermatology clinic. Thirty patients in the case group and 30 healthy controls were included in the study. Thickness of Achilles tendon enthesis was scanned by an expert rheumatologist using ultrasound equipped with a 5-14 MHz linear prob bilaterally.

**Results:**

The mean age of the patient and control groups was 43.97±16.82 years and 38.87±12.71 years, respectively (P=0.190). The mean thickness of the Achilles tendon enthesis in the dominant limb was 4.31±0.86 mm in the patient group and 4.10±0.54 mm in the control group. There was no significant difference between the two groups in terms of thickness of the Achilles tendon enthesis in the dominant limb (P=0.276). The mean thickness of the Achilles tendon enthesis in the non-dominant limb was 4.44±0.91 mm in the patient group and 4.14±0.59 mm in the control group. There was no significant difference between the two groups in terms of thickness of Achilles tendon enthesis in the non-dominant limb (P = 0.134).

**Conclusion:**

Although ultrasonography may be utilized for assessment of both structural and inflammatory changes, we revealed no difference in the mean thickness of Achilles tendon enthesis in patients with cutaneous psoriasis. Contradiction between clinical and ultrasonography features required further research.

Psoriasis is a common, chronic, immune-‌‌‌‌‌‌mediated skin disease with a wide range of clinical features. Although psoriasis is considered as a chronic disease, periods of wax and waning occur in the course of the disease. Psoriasis is found all over the world (1). However, its prevalence is noticeably different. The overall prevalence is about 2%. Psoriasis has different clinical features, and the most common type of this disease is psoriasis vulgaris which has been reported in the form of erythematous patches and silvery scales with obvious periphery (2). The scalp, elbows, knees, foreskin and limbs are more likely to be affected. Other types of the disease include drop psoriasis, flexural psoriasis, orthodromic psoriasis, and postural psoriasis that involves the palms and soles. Psoriasis arthritis (PsA) is a chronic inflammatory arthritis that affects 5-42-% of people with psoriasis disease. PsA is characterized by pain and swelling in joints. Spondylitis may occur in a minority of patients. A destructive arthropathy has also been explained in PsA. All patients with psoriasis should be screened for joint involvement. Most of the time skin manifestation precedes the onset of arthritis. However, in about 15% of cases, arthritis may develop before skin disease (3). 

Moreover, although psoriasis primarily affects the skin and joints, recent studies have reported that the disease can lead to systemic complications such as increased risk of heart disease, chronic kidney disease, cancer and depression (4). 

Inflammation of tendons and entheses, which are common features of PsA, most frequently present in lower extremities, including the Achilles tendon, plantar fascia, patellar tendon and Quadriceps tendon (5, 6). High-resolution ultrasound is becoming a routine modality in the evaluation of patients with musculoskeletal symptoms. Musculoskeletal (MSK) ultrasound (US), even in the absence of clinical arthropathy, permits detection of early disorders of tendon and enthesis to prevent long-term damage (7, 8).

 Ultrasound provides a dynamic, real time detailed high-quality image of musculoskeletal system with low cost and no harm to patients. Scans can be done while moving the region of interest and comparing structures of both sides of the body. A wide variety of articular and periarticular conditions can be evaluated and treated via guided procedures. Magnetic resonance imaging (MRI) and US both are useful tools for tendons, joints ligaments and bone surface lesions. However, US is quicker, more available, and less expensive. Moreover, using US the operator can concentrate on the area of clinical abnormality and pain and more images can be recorded to identify the exact details of injury (8, 9). This study aimed to determine the prevalence subclinical disorder of Achilles tendon in cutaneous psoriasis patients using ultrasonography. 

## Methods

This case-control study was performed on patients with cutaneous psoriasis referred to Hazrat Rasoul Akram Hospital. Inclusion criteria were patients aged 18–65 years with cutaneous psoriasis and with no self-reported history of injury to lower limbs from February 2021 to September 2021. Clinical signs of articular and periarticular involvement, systemic treatment for psoriasis during the last 6 weeks, history of musculoskeletal trauma, taking anti-inflammatory non-steroidal or glucocorticoid drugs in the last two weeks, treatment with biologic medicine, diabetes, chronic renal failure and congenital defects were considered as exclusion criteria among case and control groups. Finally, this study was conducted with 30 patients as case group and 30 healthy volunteers as control group. The Ethics Committee of Iran University of Medical Sciences approved the study (IR.IUMS.FMD.REC.1400.094). Written informed consent was obtained from all individuals who participated in the study. Achilles tendon of both limbs was examined by an experienced rheumatologist with US machine (ultrasonic, Canada) equipped with 5-14 MHz linear array transducer. Achilles tendon as a frequent site of involvement in PsA was selected. Entheseal abnormality, including bone erosions, calcifications (enthesophytes), presence of bursitis were evaluated as well. Participants lay in prone position with both knee extended and ankles were set at 90-degree flexion. Considering that the Entheseal thickness is the best indicator of tendon condition, it was measured at the site of maximal thickness 10 mm proximal to calcaneus. Data analysis was conducted using SPSS Version 26 software. To compare quantitative variables, the independent t-test or Mann-Whitney U test and for qualitative variables Chi-square or Fisher Exact’s test were used, respectively. Mean and standard deviation (SD) were used in the descriptive analyses (10). Level of significance was considered less than 0.05.

## Results

**Figure 1:**
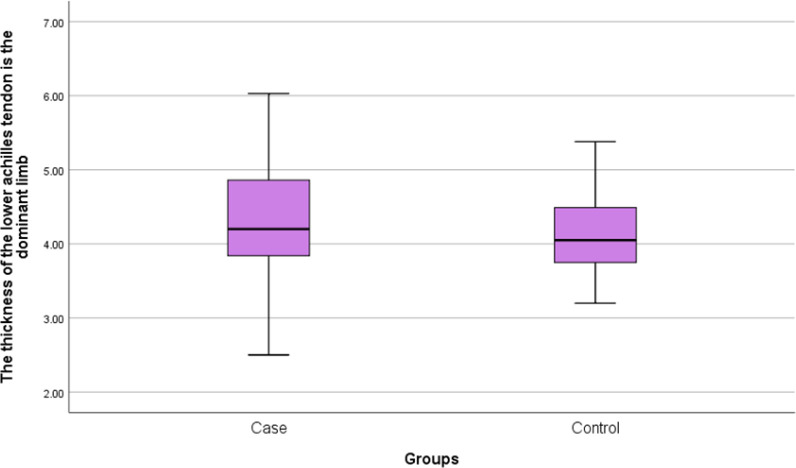
Frequency distribution of Achilles tendon’s lower part mean thickness in the dominant limb of studies participants

**Figure 2:**
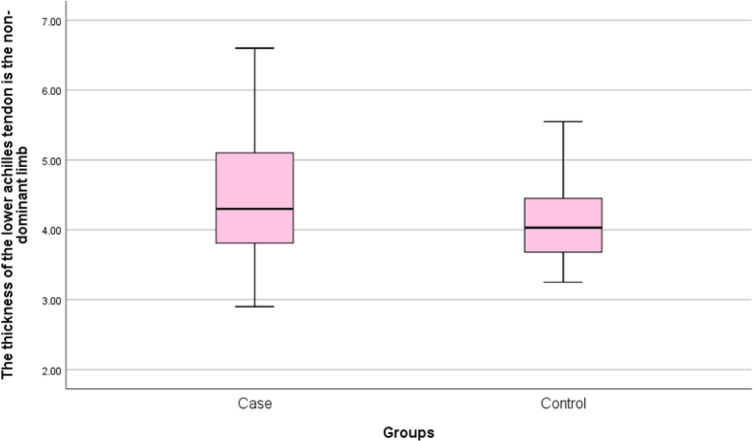
Frequency distribution of Achilles tendon’s lower part mean thickness in the non-dominant limb of studies participants

Thirty patients with cutaneous psoriasis as the patient group and 30 healthy individuals as the control group were included in this study. Mean age of patients in the case and control groups was 43.97 ± 16.82 and 38.87 ± 12.71 years, respectively. There was no significant difference between the two groups in terms of age (P=0.190). The mean weight of patients was 81.80 ±19.92 Kg in the patient group and 76.50 ± 14.54 Kg in the control group. There was no significant difference between the two studied groups in terms of weight (P=0.237). Mean body mass index (BMI) was 28.55 ± 6.17 Kg/m^2^ and 26.36 ± 4.66 Kg/m^2 ^in the patients and control groups, respectively. There was no significant difference between the two studied groups in terms of BMI (P=0.126) (table 1). Mean thickness of Achilles tendon in the lower area of the dominant limb in the patient group was 4.31 ± 0.86 mm and in the control, group was 4.10 ± 0.54 mm. There was no significant difference between the two groups in terms of thickness of Achilles tendon in the lower area in the dominant limb (P=0.276) (figure 1). Mean thickness of Achilles tendon in the lower area in non-dominant limb in the patient group was 4.44 ± 0.91 mm and in the control, group was 4.10±0.54 mm. There was no significant difference between the two groups in terms of thickness of Achilles tendon in the lower area in non-dominant limb (P=0.134) (figure 2). No bone erosion, calcification and bursitis were detected in both groups.

**Table 1:** Description of the study population

Variables						**P-Value**
	**Groups**	**N**	**Mean**	**SD**	**SEM**	
**Age (years)**	**Case**	30	43.97	16.823	3.071	0.190
	**Control**	30	38.87	12.711	2.321	
**Weight (kg)**	**Case**	30	81.80	19.493	3.559	0.237
	**Control**	30	76.50	14.543	2.655	
**Height (cm)**	**Case**	30	169.17	9.458	1.727	0.623
	**Control**	30	170.43	10.355	1.890	
**BMI (kg / m2)**	**Case**	30	28.5581	6.17011	1.12650	0.126
	**Control**	30	26.3675	4.66454	0.85162	

Independent Sample T-test is used with p<0.05 considered as significant.

**Abbreviations:** BMI: body mass index; N: number; SD: standard deviation; SEM: standard error of mean.

## Discussion

The present study was performed to determine the sonographic features of Achilles tendon in cutaneous psoriasis patients with no feature of PsA and compare it to control group. We aimed to detect evidence of subclinical enthesopathy in US images. Increased tendon or ligament thickness was the most common feature of soft tissue inflammation in 20%-57% of patients with cutaneous psoriasis. Particularly, the junction area of the Achilles tendon with the calcaneus is one of the most common sites of tendon inflammation in patients with cutaneous psoriasis and PsA (11). 

Tendon thickening may be due to edema or fibrosis. Monitoring of patients may be needed for assessment of prognosis (12). Pistone et al. reported that US could detect early inflammation of Achilles tendon before clinical signs of PsA (13).

In the early stages of tendinopathy, abnormal images of tendons detected by US or MRI may be explained by disruption of the normal fibrillar structure (14). Some studies show a significant increase in Achilles tendon thickness in cutaneous psoriasis patients compared with healthy individuals (7, 14, 15). However, Graceffa et al. showed that (16) Achilles tendon thickness at the superior pole of the calcaneus was not different between only skin psoriasis, PsA and healthy individuals, consistent with the findings of the present study. However, analysis of the inferior pole of the calcaneus, and summing the thickness of the eight entheses revealed statistically significant differences among the three groups. Moreover, tendon thickness score was correlated with areas of bone erosion in PsA patients. These findings, with data regarding the correlation between tissue thickness and calcification grade may indicate a close relationship between damage of area and tendon tissue thickness (15, 17). Monitoring of patients with US disclosed that recovery of Achilles tendonitis and retrocalcaneal bursitis occurs, while other surveys reported no improvement over time (18, 19).

Thickness measurement is the most valid method for tendonitis (20). As the doppler signal strength may not accurately detect inflammation, especially in the early stages of the disease. But findings such as calcification or erosion were identified in sonography which express a chronic inflammation (16, 21). In the present study, there was no significant difference between the two groups in terms of age, weight and BMI, which is consistent with the findings of similar study (16). 

It seems that the time of ultrasound examination can play a role in different results of studies. Also, ultrasound is a powerful instrument for evaluating medication efficacy, improvement or treatment failure. In the study of Pistone et al. (22), 30 patients with moderate to severe psoriasis undergo systemic treatment with etanercept for 72 weeks. US examination revealed thickening of Achilles tendon in 32% of cases. Doppler hypervascularity in 62%of patients. In the 12^th^ week of treatment, 53.3% of patients showed signs of improvement. At 48 and 72 weeks of treatment, no tendon thickening was detected and only chronic inflammatory consequences of enthesopathy such as calcification, erosion, and enthesophyte, remained. US is recommended as a non-invasive method in the follow-up of psoriasis patients monitoring disease activity (22). In the present study, only patients with mild to moderate skin involvement were included because patients with severe psoriasis need systemic treatment who could not be included in the study. If we could evaluate patients with severe skin involvement, we might find evidence of silent tendinopathy by ultrasound.

The most noticeable limitation of our study was insufficient sample size for statistical measurement. As the study was performed during the era of COVID-19 pandemic, many patients were reluctant to come to the hospital or participate in research studies. The other limitation was assessment of only Achilles tendon, indicative of subclinical tendinitis. Studies with larger sample size, assessment of multiple tendon sites and including patients with PsA is suggested.

Psoriatic arthritis (PsA) is an inflammatory destructive disease that its early diagnosis and treatment is of utmost importance. Clinical examinations of the tendon enthesis are clinically challenging; thus, ultrasonography may be used as a reliable imaging tool for assessment of both structural and inflammatory changes. However, we did not indicate a significant difference in the mean thickness of Achilles tendon enthesis in patients with cutaneous psoriasis. There is contradictory association between clinical and ultrasonography findings; thus, further research is required to elucidate this discordance.

## References

[1] Tashiro T, Sawada Y (2022). Psoriasis and systemic inflammatory disorders. Int J Mol Sci.

[2] Meer E, Thrastardottir T, Wang X (2022). Risk factors for diagnosis of psoriatic arthritis, psoriasis, rheumatoid arthritis, and ankylosing spondylitis: a set of parallel case-control studies. J Rheumatol.

[3] Azuaga AB, Ramírez J, Cañete JD (2023). Psoriatic arthritis: pathogenesis and targeted therapies. Int J Mol Sci.

[4] Jin JQ, Elhage KG, Spencer RK (2023). Mendelian randomization studies in psoriasis and psoriatic arthritis: A systematic review. J Invest Dermatol.

[5] Chen YJ, Chang YT, Shen JL (2012). Association between systemic antipsoriatic drugs and cardiovascular risk in patients with psoriasis with or without psoriatic arthritis: a nationwide cohort study. Arthritis Rheum.

[6] Chisălău BA, Bărbulescu AL, Pârvănescu CD (2021). Entheseal involvement in a group of psoriatic arthritis patients: An ultrasonographic study. Exp Ther Med.

[7] Gisondi P, Tinazzi I, El-Dalati G (2008). Lower limb enthesopathy in patients with psoriasis without clinical signs of arthropathy: a hospital-based case-control study. Ann Rheum Dis.

[8] Dubash SR, De Marco G, Wakefield RJ (2020). Ultrasound imaging in psoriatic arthritis: what have we learnt in the last five years?. Front Med (Lausanne).

[9] Morvan G, Vuillemin V, Guerini H (2012). Interventional musculoskeletal ultrasonography of the lower limb. Diagn Interv Imaging.

[10] Seif F, Khoshmirsafa M, Mousavi M, Beshkar P, Rafeian-Kopaei M, Bagheri N, Shirzad H (2014). Interleukin-21 receptor might be a novel therapeutic target for the treatment of rheumatoid arthritis. Journal of Experimental & Clinical Medicine..

[11] Simon D, Tascilar K, Kleyer A (2020). Structural entheseal lesions in patients with psoriasis are associated with an increased risk of progression to Vy psoriatic arthritis. Arthritis Rheumatol.

[12] Van Der Ven M, Karreman MC, Weel AE (2016). Adding ultrasound to clinical examination reduced frequency of enthesitis in primary care psoriasis patients with musculoskeletal complaints. Clin Exp Rheumatol.

[13] Pistone G, La Vecchia M, Pistone A, Bongiorno M R (2014). Achilles tendon ultrasonography may detect early features of psoriatic arthropathy in patients with cutaneous psoriasis. Br J Dermatol.

[14] McGonagle D, Marzo-Ortega H, O'connor P (2002). Histological assessment of the early enthesitis lesion in spondyloarthropathy. Ann Rheum Dis.

[15] Vyas K, Jain SK, Mittal A (2020). Sonographic evaluation of subclinical enthesopathy in patients of chronic plaque psoriasis. Indian Dermatol Online J.

[16] Graceffa D, Bonifati C, Lora V (2019). Sectional study. Indian J Dermatol Venereol Leprol..

[17] Bandinelli F, Prignano F, Bonciani D (2013). Ultrasound detects occult entheseal involvement in early psoriatic arthritis independently of clinical features and psoriasis severity. Clin Exp Rheumatol.

[18] Macchioni P, Salvarani C, Possemato N (2019). Ultrasonographic and clinical assessment of peripheral enthesitis in patients with psoriatic arthritis, psoriasis, and fibromyalgia syndrome: The ULISSE Study. J Rheumatol.

[19] Tang Y, Yang Y, Xiang X (2018). Power Doppler ultrasound evaluation of peripheral joint, entheses, tendon, and bursa abnormalities in psoriatic patients: A clinical study. J Rheumatol.

[20] Graceffa D, Bonifati C, Lora V (2019). Ultrasound assessment of enthesis thickness in psoriasis and psoriatic arthritis: A cross-sectional study. Indian J Dermatol Venereol Leprol.

[21] Eder L, Jayakar J, Thavaneswaran A (2014). Is the MAdrid Sonographic Enthesitis Index useful for differentiating psoriatic arthritis from psoriasis alone and healthy controls?. J Rheumatol.

[22] Pistone G, Gurreri R, Bongiorno MR (2016). Assessment of etanercept efficacy in the treatment of psoriatic arthritis: ultrasonography of Achilles tendon. J Eur Acad Dermatol Venereol.

